# Curve Progression in Adolescent Idiopathic Scoliosis with Cobb Angles Between 40 and 50 Degrees at the Late Stage of Skeletal Growth: A Minimum 5-Year Follow-Up Study

**DOI:** 10.3390/jcm14155272

**Published:** 2025-07-25

**Authors:** Yunjin Nam, Udit Patel, Dong-Gune Chang, Young Bin Lee, Jungwook Lim, Jae Hyuk Yang, Seung Woo Suh

**Affiliations:** 1 Department of Orthopedic Surgery, Korea University Guro Hospital, Seoul 08308, Republic of Korea; nam.yunjin@gmail.com (Y.N.); uditpatel27@gmail.com (U.P.); ys98194@gmail.com (Y.B.L.); jlim2012@gmail.com (J.L.); 2 Department of Orthopedic Surgery, Inje University Sanggye Paik Hospital, Seoul 01757, Republic of Korea; dgchangmd@gmail.com; 3 Department of Orthopedic Surgery, Korea University Anam Hospital, Seoul 02841, Republic of Korea; kuspine@naver.com

**Keywords:** adolescent idiopathic scoliosis, skeletal maturity, progression, risk factor

## Abstract

**Background/Objectives**: Surgical treatment is generally recommended for adolescent idiopathic scoliosis (AIS) when the Cobb angle exceeds 50 degrees even after skeletal maturity or 40 degrees with remaining growth potential. However, limited evidence exists regarding the natural history of curves between 40 and 50 degrees during the late stage of skeletal growth. This study aimed to evaluate the curve progression in AIS patients with a curve between 40 and 50 degrees at Risser stage IV or V. **Methods**: The inclusion criteria were as follows: (1) AIS patients at the late stage of skeletal growth (Risser IV or V) and a (2) curve between 40 and 50 degrees, with a minimum follow-up of 5 years. Sex, age, the magnitude of the curve, the location of the apex, Risser stage, height, and weight were measured at the baseline and the final follow-up. Curve progression was defined as an increase in the Cobb angle of ≥5 degrees. Patients were also categorized based on whether their final Cobb angle was <50 or ≥50 degrees to evaluate additional risk factors. **Results**: A total of 97 patients were included, with a mean follow-up of 97 months. Their mean age was 14.6 years at the baseline and 22.6 years at the final follow-up. The mean Cobb angle increased from 42.6 to 45.1 degrees, with a mean change of 2.7 degrees and an annual progression rate of 0.35 degrees. Curve progression was observed in 38 patients (39.2%), and 24 patients (24.7%) reached a final Cobb angle ≥ 50 degrees. Younger age (*p* = 0.004) and Risser stage IV (*p* = 0.014) were significantly associated with curve progression. In patients with a final Cobb angle ≥ 50 degrees, Risser stage IV (*p* = 0.050) and a larger baseline curve magnitude (*p* = 0.045) were also significant risk factors. **Conclusions**: In AIS patients at the late stage of skeletal growth, 39.2% experienced significant curve progression. A younger age and Risser stage IV were identified as risk factors for curve progression. A larger baseline curve magnitude and Risser stage IV were also associated with a final Cobb angle ≥ 50 degrees.

## 1. Introduction

Adolescent idiopathic scoliosis (AIS) is a three-dimensional spinal deformity that most commonly develops during early adolescence [1]. Although AIS is often asymptomatic, surgical treatment is generally recommended when the Cobb angle exceeds 50 degrees due to concerns about pulmonary dysfunction, impaired spinal function, and cosmetic deformity [2,3]. Consequently, numerous studies have investigated the risk factors associated with curve progression [4].

Curve progression in AIS is typically defined as an increase of 5 to 10 degrees in the Cobb angle on serial radiographs. Key determinants of progression include skeletal maturity, chronological age, sex, growth potential, and initial curve magnitude. Despite the availability of radiographic parameters, accurately predicting curve progression remains challenging due to the multifactorial nature of and highly variable growth patterns in AIS [5,6,7].

Weinstein et al. [8,9] and Nachemson et al. [10] emphasized the importance of age and curve size in predicting progression, demonstrating a strong correlation between these factors and long-term outcomes. Weinstein et al. [8,9] and Ascani et al. [11] further reported that curves under 30 degrees at skeletal maturity rarely progress during adulthood, whereas curves exceeding 50 degrees tend to progress at an average rate of approximately 1 degree per year. However, the natural history of curves measuring between 40 and 50 degrees, particularly during the late stages of skeletal growth, remains insufficiently understood.

Although skeletal maturity is classically defined as Risser stage V, many clinical protocols regard Risser stage IV as an appropriate time to wean bracing, based on the assumption that growth is nearing completion [12,13]. However, recent studies have reported that residual growth may persist beyond Risser stage IV in some patients, potentially contributing to further curve progression despite brace weaning [14]. Thus, the late stage of skeletal growth, encompassing Risser stages IV and V, represents a transitional but clinically significant period for decision-making regarding conservative treatment discontinuation and surgical indications.

The present study aimed to evaluate the rate of curve progression in AIS patients with Cobb angles between 40 and 50 degrees during this late stage of skeletal growth and to identify risk factors for curve progression.

## 2. Materials and Methods

This study was a single-center, retrospective study based on a medical record review. A total of 97 patients were identified from the Scoliosis Research Institute database between 1999 and 2016. The inclusion criteria were as follows: (a) a diagnosis of adolescent idiopathic scoliosis (AIS); (b) a major curve Cobb angle between 40 and 50 degrees at the late stage of skeletal growth; (c) Risser stage ≥ IV; and (d) a minimum of 5 years of follow-up without any prior surgical intervention for AIS. The time point at which both criteria (b) and (c) were first met was defined as the baseline, and the time of the last available radiographic follow-up was defined as the final follow-up. For patients who eventually underwent surgery after the 5-year follow-up period, the final follow-up was defined as the last radiograph taken prior to surgery.

At our institution, AIS patients with curves between 40 and 50 degrees at the late stage of skeletal growth are routinely offered annual or more frequent follow-ups. Skeletal maturity was determined using the American Risser staging system, in which ossification and fusion of the iliac apophysis are graded from stage 0 to V. A Risser stage ≥ IV was considered the late stage of skeletal growth. Radiographic evaluation of the Risser stage was based on whole-spine posteroanterior (PA) radiographs and pelvic inlet views.

In this study, a Cobb angle increase of <5 degrees after at least 5 years of follow-up was considered ‘no curve progression’, and a Cobb angle increase of ≥5 degrees in the major curve was considered ‘curve progression’. The rate of curve progression was calculated as the absolute change in the Cobb angle divided by the follow-up duration in years. Patients were also categorized into two groups based on whether their final Cobb angle was <50 degrees or ≥50 degrees at the final follow-up. A risk factor analysis was performed separately for two outcomes: (1) curve progression defined as an increase of ≥5 degrees during follow-up, and (2) a final Cobb angle ≥ 50 degrees at the final follow-up.

The baseline measurements included sex, age, curve magnitude, apex location, Lenke classification, Risser stage, height, and weight. The same parameters, excluding sex, apex location, and Lenke classification, were recorded again at the final follow-up. Changes in curve magnitude, height, weight, and the rate of curve progression per year were also calculated.

The data analysis was performed using IBM SPSS Statistics for Windows, version 20.0.0 (IBM Corp., Armonk, NY, USA). Student’s t-test was used to analyze continuous variables, and the chi-squared test was used to analyze categorical variables. *p*-values ≤ 0.05 were considered statistically significant.

## 3. Results

A total of 97 patients (74 females, 23 males) were included. The mean age at the baseline was 14.6 ± 1.7 years (range: 9–19), and the mean follow-up period was 97 months (range: 61–245). At the final follow-up, the mean age was 22.6 ± 3.2 years (range: 18–35). At the baseline, 65 patients were Risser stage IV, and 32 were Risser stage V. At the final follow-up, 96 patients were Risser stage V, and 1 patient remained at stage IV. Among the total cohort, six patients (6.2%) underwent surgical treatment at a mean of 98.5 months (range: 79–183) after the 5-year follow-up period.

The mean Cobb angle was 42.6 degrees (range: 40–49) at the baseline and 45.1 degrees (range: 29–68) at the final follow-up, with a mean change of 2.7 ± 6.5 degrees (range: −12 to 28). The mean annual progression rate was 0.35 ± 0.90 degrees per year (range: −2.12 to 2.82).

Among the 97 patients, 38 (39.2%) showed curve progression, while 59 (60.8%) did not. In the no-progression group, the mean age at the baseline was 15.0 ± 1.8 years, compared to 13.9 ± 1.2 years in the progression group (*p* = 0.001). In the no-progression group, 34 patients were Risser stage IV and 25 were Risser stage V, whereas in the progression group, 31 patients were Risser stage IV and 7 were Risser stage V (*p* = 0.014). The baseline Cobb angle was similar between the two groups: 42.6 ± 2.7 degrees in the no-progression group and 42.5 ± 2.7 degrees in the progression group (*p* = 0.841) (Table 1). In the progression group, the mean increase in the Cobb angle was 9.1 degrees, with an average progression rate of 1.2 degrees per year.

Among the 38 patients with progression, 24 (24.7%) reached a Cobb angle of ≥50 degrees at the final follow-up. In the group with a final Cobb angle <50 degrees, there were 55 females and 18 males; in the ≥50-degree group, 19 were female, and 5 were male. The mean age at the first visit was 14.7 ± 1.7 years in the <50-degree group and 14.2 ± 1.3 years in the ≥50-degree group (*p* = 0.168). In the <50-degree group, 45 patients were Risser stage IV and 28 were Risser stage V, while in the ≥50-degree group, 20 were Risser stage IV and 4 were Risser stage V (*p* = 0.050). The mean baseline Cobb angle was 42.3 ± 2.6 degrees in the <50-degree group and 43.6 ± 2.9 degrees in the ≥50-degree group (*p* = 0.045) (Table 2). In patients with a final Cobb angle ≥ 50 degrees, the mean progression was 10.3 degrees, with an annual progression rate of 1.3 degrees.

Between the progression and no-progression groups, significant differences were found in age at the baseline (13.9 vs. 15.0 years, *p* = 0.004) and the Risser stage distribution (stage IV vs. V, *p* = 0.014). Between the groups with a final Cobb angle < 50 degrees and ≥50 degrees, Risser stage (*p* = 0.050) and baseline curve magnitude (*p* = 0.045) showed statistically significant differences.

**Figure 1:**
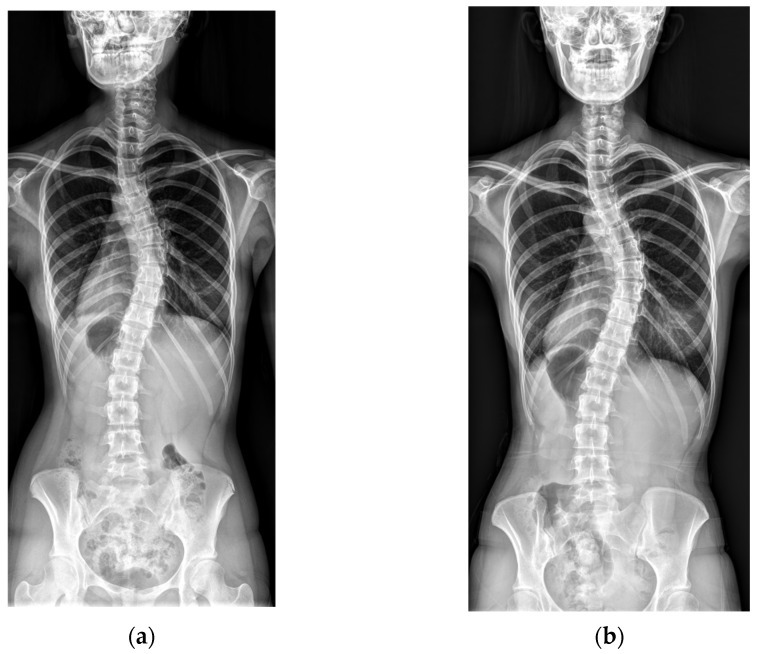
An example of no curve progression. (**a**) A baseline radiograph of a 16-year-old female patient with Risser stage V and a Cobb angle of 41°. (**b**) A final radiograph at age 22, showing Risser stage V and a Cobb angle of 43°.

**Figure 2:**
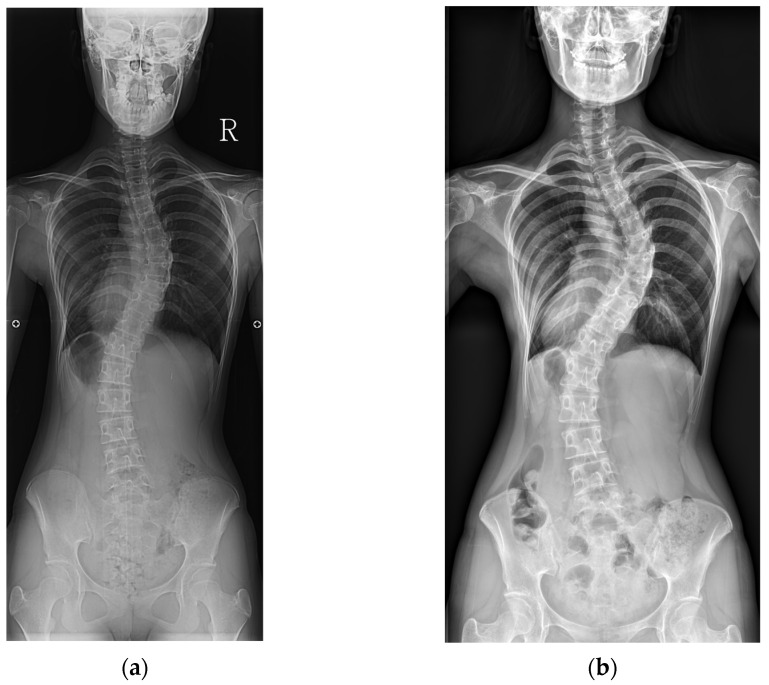
An example of curve progression. (**a**) A baseline radiograph of a 14-year-old female patient with Risser stage IV and a Cobb angle of 44°. (**b**) A final radiograph at age 26, showing Risser stage V and a Cobb angle of 62°.

Representative cases from the no-progression and progression groups are shown in Figure 1 and Figure 2.

## 4. Discussion

Progression of the scoliotic curve is a primary indication for surgical intervention in AIS. Approximately 10% of AIS cases progress to a degree requiring surgery [15]. However, predicting curve progression is still a clinical challenge. In general, the risk of progression increases with curve magnitude and the amount of remaining skeletal growth. Skeletal maturity and initial curve magnitude are considered the two most important variables when determining whether conservative or surgical treatment is appropriate. Skeletal maturity should be carefully assessed, as the risk of curve progression increases in younger patients and those with larger initial curve magnitudes [7,16,17].

Although it is well established that curves over 50 degrees progress after skeletal maturity [8,18], AIS curves between 40 and 50 degrees after the late stage of skeletal growth represent a critical therapeutic gray zone. These curves are often considered close to the surgical threshold but do not always warrant immediate intervention. Understanding their natural history is essential, particularly in the absence of bracing or surgery.

Ward et al. [19] reported that in patients with Risser stage IV or V, curves measuring between 40 and 50 degrees showed progression patterns comparable to those for curves ≥ 50 degrees. Consistently, Ascani et al. [11] reported that curves can continue to progress after skeletal maturity, particularly larger curves. In their study, thoracic curves between 40 and 50 degrees showed progression patterns similar to those for curves exceeding 50 degrees. Similarly, Yu et al. [20] reported that 61.6% of patients with curves between 40 and 50 degrees experienced progression after skeletal maturity. Although their study included some patients who did not meet the criteria for absolute skeletal maturity, such as those at Risser stage IV, it nonetheless suggested that curves exceeding 40 degrees may still progress during the late stage of skeletal growth. In our study, 39.2% of the curves in the 40- to 50-degree range progressed after the late stage of skeletal growth, and even among those with confirmed skeletal maturity, 21.9% showed progression. These findings collectively emphasize the need for close monitoring even in patients who appear to be skeletally mature.

Bone age is an important predictor of curve progression, as it reflects skeletal maturity. Several methods have been developed to assess bone age. Ward et al. [19] demonstrated that Risser stage was significantly associated with both the absolute and annualized curve progression over a 10-year follow-up. Although Yu et al. [20] used the distal radius and ulna classification to evaluate bone age in their study, they also utilized Risser stage in their analysis. Collis et al. [17] reported post-maturity curve progression in 27% of patients at Risser stage IV and 28.4% at stage V. However, many studies have grouped Risser stages IV and V together as a single category of skeletal maturity. While Risser stage IV clearly represents the later part of skeletal growth, it does not indicate the complete cessation of growth and should be distinguished from Risser stage V. In our study, curve progression was observed in 47.7% of the patients at Risser stage IV, compared to 21.9% of those at Risser stage V (*p* = 0.014). Furthermore, 30.8% of the patients at Risser stage IV progressed to a final Cobb angle ≥ 50 degrees, while only 12.5% of those at Risser stage V did (*p* = 0.050). These findings suggest that Risser stage IV, although indicative of decelerating growth, does not equate to skeletal maturity and should be considered separately from stage V. Previous studies that included Risser stage IV as part of skeletal maturity may have overestimated the risk of curve progression. At the same time, our results also demonstrate that curve progression, although less frequent, can still occur in patients who have reached Risser stage V and are considered skeletally mature.

Recent evidence suggests that curve progression can still occur beyond Risser stage IV, and bracing may remain beneficial even at this late skeletal stage, particularly in patients with residual growth or documented progression. According to a systematic review by Zaina et al. [21], bracing can be effective even in patients with curves ≥ 40 degrees who are skeletally immature or have ongoing progression. This supports the notion that curves between the 40- and 50-degree range at Risser stage IV represent a therapeutic window during which bracing may still be appropriate. Moreover, long-term follow-up remains important even after skeletal growth is nearly complete. In our study, 24 of 97 patients (24.7%) progressed to a Cobb angle of 50 degrees or more during the follow-up period. This is a clinically significant finding, as 50 degrees is widely regarded as the threshold for surgical consideration. Although these patients were already in the late stage of skeletal maturity at the start of this study, they experienced meaningful curve progression within five years. Notably, patients at Risser stage IV and those with larger initial curves were more likely to exceed the 50-degree threshold. Although bracing had been discontinued, our findings suggest that patients with greater initial Cobb angles may still require close monitoring. These results highlight the importance of personalized follow-up strategies, even after bracing has ended. Therefore, while this study provides insights into the natural history of untreated AIS in patients at the late stage of skeletal growth, it also highlights the importance of individualized management strategies, which may include bracing even after the conventional weaning thresholds. In our study, 24 patients experienced curve progression to 50 degrees or more, but only 6 of them ultimately underwent surgical treatment. The mean final Cobb angle among the patients who underwent surgery was 54.8 degrees, which was not significantly different from the mean of 53.8 degrees in the subgroup with curves of 50 degrees or more. This finding suggests that surgical decisions are influenced by various factors, including symptoms, curve pattern, patient preferences, and quality of life, and are not based solely on curve magnitude.

Chronological age is also a well-established risk factor for curve progression given its close association with physical growth, skeletal development, and the onset of menarche in female patients. In a study of 1,464 patients, Lee et al. [22] reported that the risk of progression was higher when the age at presentation was under 11.3 years. In our study, the mean age at the baseline was 13.9 ± 1.2 years in the progression group and 15.0 ± 1.8 years in the non-progression group, showing a significant difference of approximately 1.1 years (*p* = 0.001). These findings suggest that younger chronological age is associated with a higher risk of curve progression, underscoring the importance of evaluating both bone age and chronological age rather than relying solely on skeletal maturity. Chronological age emerged as the most statistically significant factor in our analysis. The mean age at the final follow-up was 22.6 years, with most patients being under the age of 30, indicating that the results of this study should be interpreted within the context of a relatively young adult population and may not be generalizable to middle-aged or elderly individuals with AIS.

Initial curve magnitude has consistently been reported as a strong predictor of progression. Lee et al. [22] identified it as the most critical determinant among 1464 patients. Similarly, Lara et al. [23] found that initial curve size was a significant risk factor for curve progression. In our study, the baseline curve magnitude was restricted to a narrow range between 40 and 50 degrees, which has generally been regarded to represent a similar level of curve severity in previous studies. Among the patients with curve progression, the mean initial curve magnitude was 42.6 ± 2.7 degrees, while it was 42.5 ± 2.7 degrees in those without progression, showing no significant difference between the two groups. However, when comparing patients based on whether their final curve reached 50 degrees, there was a statistically significant difference in the baseline curve magnitude: 43.6 ± 2.9 degrees in the ≥50-degree group versus 42.3 ± 2.6° in the <50-degree group (*p* = 0.045). When stratifying the initial curves into two subgroups, 40 to 45 degrees (n = 74) and 45 to 50 degrees (n = 23), there was no significant association with curve progression (*p* = 0.846), but there was a significant difference in the likelihood of the final curve reaching 50 degrees or greater (*p* = 0.027). This is likely due to the definition of progression as an increase of more than 5 degrees, meaning that curves starting at 45 degrees or above could surpass 50 degrees even with minimal progression. Since a Cobb angle of 50 degrees or greater is often considered a surgical threshold, curves measuring 45 degrees or more at presentation should be monitored more closely due to their higher potential to exceed this threshold, even with minor progression.

Recent studies have developed prediction models to improve our understanding of curve progression during the growth period. For instance, Parent et al. [24] demonstrated that prior radiographs can be used to predict future curve angles in untreated AIS patients, from age 6 through to the end of growth. Negrini et al. [25] further emphasized that puberty significantly alters the natural history of scoliosis and proposed growth-stage-specific prediction models. Additionally, Dolan et al. [26] validated a prognostic model based on the Simplified Skeletal Maturity System for identifying high-risk patients before skeletal maturity. While our study focused on progression after the late stage of skeletal growth, these prediction models offer important context for identifying which patients may benefit from early interventions or closer follow-up. Incorporating such tools into clinical decision-making may help refine the treatment strategies, especially for AIS curves in the therapeutic gray zone between 40 and 50 degrees.

In our cohort, the location of the main curvature was analyzed based on the apex, which was categorized as thoracic, thoracolumbar, or lumbar. Although the differences did not reach statistical significance, patients with thoracic curves demonstrated relatively higher rates of progression (44.6%) and reaching a Cobb angle of 50 degrees or more (29.7%) compared to those for other curve locations. This pattern is consistent with the findings of Yu et al. [20], who also reported that thoracic curves were more prone to progression after skeletal maturity. These findings suggest that thoracic curves, even in skeletally mature patients, may require particularly close monitoring. Additionally, the curve types were classified based on the Lenke classification. However, no statistically significant differences in curve progression were found among the six Lenke types. This may be due to the relatively small number of patients in each Lenke subgroup, which limited the statistical power to detect meaningful differences. Although not conclusive, these trends indicate that curve morphology, including apex location and Lenke type, may influence progression risk and should be investigated further in larger prospective studies.

This study has several limitations. As a retrospective study, it is subject to selection bias. We excluded patients who underwent surgery during the follow-up period, which may have led to an underestimation of the true progression rate and the natural history of AIS curves between 40 and 50 degrees. In our study, we excluded only those who underwent surgery within the 5-year follow-up period, as their progression status could not be consistently evaluated over the required observation window. Specifically, three patients who initially met the inclusion criteria (Risser stage ≥ IV and a Cobb angle between 40 and 50 degrees) underwent surgery at 13, 33, and 47 months, respectively. All three patients showed a Cobb angle progression ≥ 5 degrees and reached ≥50 degrees before surgery. Importantly, patients who underwent surgery after the 5-year follow-up period were not excluded. Among the 97 patients included in the final cohort, 6 eventually received surgical treatment beyond 5 years, and their last preoperative follow-up was used as their final data point for analysis. Therefore, the exclusion was limited to 3 cases out of approximately 100 screened patients, minimizing the magnitude of bias. This methodological decision was made to ensure a consistent assessment of progression over a fixed follow-up period while maintaining transparency. Another limitation of this study is that although the curve types were classified based on the Lenke system, sagittal thoracic modifiers were not assessed. As sagittal alignment may influence curve progression and treatment decision-making, future prospective studies should incorporate standardized sagittal parameter measurements to clarify their role in this patient population. Additionally, the sample size in each subgroup was relatively small. Nevertheless, the results provide valuable insights and can serve as a basis for larger, multicenter studies.

## 5. Conclusions

In this study, significant curve progression was observed in 39.2% of the AIS patients even at the late stage of skeletal growth. A younger age and Risser stage IV were significantly associated with this progression. In addition, a larger baseline curve magnitude and Risser stage IV were associated with a final Cobb angle ≥ 50 degrees. Understanding these risk factors may aid clinicians in developing more appropriate management strategies for AIS patients at the late stage of skeletal growth. Given that curve progression may continue even after skeletal maturity, especially in patients with residual growth or a larger baseline curve magnitude, extended follow-up and timely clinical decision-making may help prevent progression beyond the surgical threshold.

## Figures and Tables

**Table 1:** Factors associated with curve progression.

Variable	Progression (−)	Progression (+)	*p*-Value
Sex (female/male)	44/15	30/8	0.621
Age (baseline) (years)	15.0 ± 1.8	13.9 ± 1.2	0.001 *
Height (baseline) (cm)	163.8 ± 8.4	162.7 ± 5.7	0.451
Weight (baseline) (kg)	52.4 ± 12.1	52.2 ± 9.4	0.945
Height (amount of change) (cm)	1.6 ± 1.3	1.5 ± 1.4	0.872
Weight (amount of change) (cm)	2.7 ± 3.8	1.7 ± 5.4	0.316
Follow-up period (months)	93.9 ± 33.9	96.5 ± 33.9	0.710
Location of apex (T/T-L/L)	41/8/10	33/3/2	0.128
Lenke classification (1/2/3/4/5/6)	26/0/12/4/10/7	17/0/9/6/2/4	0.339
Risser stage (IV/V)	34/25	31/7	0.014 *
Baseline curve magnitude (degrees)	42.6 ± 2.7	42.5 ± 2.7	0.841

T, thoracic; T-L, thoracolumbar; L, lumbar. * indicates statistical significance (*p*-value ≤ 0.05).

**Table 2:** Factors associated with a final Cobb angle ≥ 50 degrees.

Variable	<50 Degrees at Final	≥50 Degrees at Final	*p*-Value
Sex (female/male)	55/18	19/5	0.702
Age (baseline) (years)	14.7 ± 1.7	14.2 ± 1.3	0.168
Height (baseline) (cm)	163.7 ± 7.6	162.5 ± 6.7	0.511
Weight (baseline) (kg)	53.4 ± 12.1	49.3 ± 6.5	0.121
Height (amount of change) (cm)	1.6 ± 1.3	1.5 ± 1.5	0.741
Weight (amount of change) (cm)	2.5 ± 3.5	1.7 ± 6.7	0.490
Follow-up period (months)	93.2 ± 31.9	100.1 ± 39.2	0.390
Location of apex (T/T-L/L)	52/9/12	22/2/0	0.075
Lenke classification (1/2/3/4/5/6)	32/0/16/7/10/8	11/0/5/3/2/3	0.959
Risser stage (IV/V)	45/28	20/4	0.050 *
Baseline curve magnitude (degrees)	42.3 ± 2.6	43.6 ± 2.9	0.045 *

T, thoracic; T-L, thoracolumbar; L, lumbar. * indicates statistical significance (*p*-value ≤ 0.05).

## Data Availability

The data underlying this article cannot be shared publicly because of the privacy of the individuals who participated in this study. The data can be shared by the corresponding authors upon reasonable request.
